# Synthesis of Mixed Cu/Ce Oxide Nanoparticles by the Oil-in-Water Microemulsion Reaction Method

**DOI:** 10.3390/ma9060480

**Published:** 2016-06-16

**Authors:** Kelly Pemartin-Biernath, Andrea V. Vela-González, Maira B. Moreno-Trejo, César Leyva-Porras, Iván E. Castañeda-Reyna, Isaías Juárez-Ramírez, Conxita Solans, Margarita Sánchez-Domínguez

**Affiliations:** 1 Institut de Química Avançada de Catalunya (IQAC-CSIC) y CIBER en Biotecnología, Biomateriales y Nanomedicina (Ciber-BBN), Jordi Girona 18-26, Barcelona 08034, Spain; kelly.pemartin@gmail.com or kelly.pemartin@mcbride.eu (K.P.-B.); conxita.solans@iqac.csic.es (C.S.); 2 Mc Bride Chemolux S.A.R.L, Rue de I’Industrie, Foetz 3895, Luxembourg; 3 Centro de Investigación en Materiales Avanzados, S. C. (CIMAV), Unidad Monterrey, Alianza Norte 202, Parque de Investigación e Innovación Tecnológica, Apodaca 66628, Mexico; andrea.vela@cimav.edu.mx (A.V.V.-G.); maira.moreno@cimav.edu.mx (M.B.M.-T.); cesar.leyva@cimav.edu.mx (C.L.-P.); 4 Universidad Autónoma de Nuevo León, Facultad de Ingeniería Civil, Departamento de Ecomateriales y Energía. Cd. Universitaria, San Nicolás de los Garza 66455, Mexico; fime.iecr@live.com.mx (I.E.C.-R.); isaias.juarezrm@uanl.edu.mx (I.J.-R.)

**Keywords:** mixed oxide nanoparticles, microemulsion, nanoreactor, CeO_2_ NPs, CuO NPs

## Abstract

Cerium oxide and mixed Cu/Ce oxide nanoparticles were prepared by the oil-in-water (O/W) microemulsion reaction method in mild conditions. The Cu/Ce molar ratio was varied between 0/100 and 50/50. According to X-ray diffraction (XRD), below 30/70 Cu/Ce molar ratio, the materials presented a single phase consistent with cubic fluorite CeO_2_. However, above Cu/Ce molar ratio 30/70, an excess monoclinic CuO phase in coexistence with the predominant Cu/Ce mixed oxide was detected by XRD and High-Resolution Transmission Electron Microscopy (HRTEM). Raman spectroscopy showed that oxygen vacancies increased significantly as the Cu content was increased. Band gap (*E*_g_) was investigated as a function of the Cu/Ce molar ratio, resulting in values from 2.91 eV for CeO_2_ to 2.32 eV for the mixed oxide with 30/70 Cu/Ce molar ratio. These results indicate that below 30/70 Cu/Ce molar ratio, Cu^2+^ is at least partially incorporated into the ceria lattice and very well dispersed in general. In addition, the photodegradation of Indigo Carmine dye under visible light irradiation was explored for selected samples; it was shown that these materials can remove such contaminants, either by adsorption and/or photodegradation. The results obtained will encourage investigation into the optical and photocatalytic properties of these mixed oxides, for widening their potential applications.

## 1. Introduction

Ceria and ceria-based nanomaterials are well known for their efficiency in numerous applications, such as solid-state electrolytes for electrochemical devices [1], ultraviolet absorbents for sunscreens [2], oxygen storage [3], hybrid solar cells [4], and luminescent materials for violet/blue fluorescence [5]. Moreover, due to the excellent oxygen buffering property of ceria, they present also a high interest for heterogeneous catalysis. The most important catalytic reactions using ceria-based nanomaterials are reduction of NO [6,7], CO oxidation [8], and Water Gas Shift [9] reactions.

In many cases, the redox properties and chemical activity of pure ceria can be enhanced by introducing different types of metals (e.g., Zr, Ca, Cu, Tb, Mn, *etc.*) into the oxide [10,11,12]. At a structural level, a dopant or second metal can introduce stress into the lattice of an oxide host, inducing the formation of defects with a high chemical activity. On the other hand, the lattice of the oxide host can impose non-typical coordination modes on the dopant element with a subsequent perturbation in the dopant chemical properties. Finally, metal-metal or metal-oxygen-metal interactions in mixed-metal oxides can lead to electronic properties not seen in single-metal oxides [13]. Also, according to the dopant nature a different effect on the formation of oxygen vacancies in ceria occurs which may lead to tunable performance of the final mixed oxide.

With the challenge of replacing the expensive and scarcely available noble metal catalysts, scientists have been encouraged to use transition metal oxide nanoparticles. Recently, it has been shown that CeO_2_-CuO mixed oxide nanomaterials were promising nanocatalysts for the preferential oxidation of CO [14]. Copper oxide nanomaterials are applied in various areas: gas sensors [15], biosensors [16], batteries [17], high temperature superconductors [18], solar energy conversion [19], field emission emitters [20], and catalysis [21]. It has been reported that the efficiency of CeO_2_-CuO nanocatalysts could be related to synergetic interactions between CeO_2_ and CuO promoting special redox properties at their interface [22]. However, it is worth noting that the redox properties of the obtained mixed oxides depend on the preparation methods promoting different catalytic performance due to the various states of dispersion of copper [23].

CeO_2_-CuO nanoparticles have been prepared by several methods. For example, the material synthesized by the hydrothermal method presented a high performance for selective oxidation of CO in excess H_2_ [24]. In addition, Szabova *et al.* reported another method that consists of the deposition of metallic Cu onto a previously prepared CeO_2_ surface in 12 consecutive steps [25]. Gurbani *et al.* reviewed other methods to prepare Cu-Ce mixed oxides, such as co-precipitation, sol-gel, and urea-nitrate combustion [26]. The main disadvantages of the cited methods are the requirements of high temperature (min. 80 °C) for several hours, involving high energy input and/or sophisticated techniques or equipment. In order to overcome this environmental impact, a new promising method for the synthesis of metal and metal oxide nanoparticles was recently developed, based on the use of oil-in-water (O/W) microemulsions as confined reaction media [27]. In addition to the advantages of the traditional water-in-oil (W/O) microemulsion method [28,29,30], namely, a high control of particle size, a high purity, and good chemical homogeneity under mild conditions, the O/W microemulsion method has the advantage of requiring lower organic solvent concentrations, as the predominant component is aqueous [27,31]. The O/W microemulsion reaction method, firstly reported by our research group in 2009 [27] for the preparation of metal and metal oxide nanoparticles, consists of the use of organometallic precursors, dissolved within nanometer scale oil droplets (stabilized by surfactant), and dispersed in a continuous aqueous phase. The thermodynamic stability of microemulsions maintains the oil phase (carrying the metal precursors) and the aqueous phase (containing the precipitating agent) intimately mixed leading to an increased oil-water interface. The confined media at the nanometer level created by the microemulsion environment may lead to an increased synergy between oxides at their interface which may result in better properties and improved performance.

The present work reports on the synthesis of mixed Cu/Ce oxide nanoparticles using the mild oil-in-water microemulsion reaction method. Characteristics of the as-obtained and calcined nanomaterials were compared as a function of Cu/Ce molar ratio in order to determine the maximum Cu content that could be incorporated into the CeO_2_ crystal lattice with a good Cu dispersion, which is a relevant characteristic in catalytic applications. Magnetic, optical (*E*_g_ as a function of Cu content), and photocatalytic properties are presented.

## 2. Results

### 2.1. Microemulsion Formulation and Synthesis of Cu/Ce Nanoparticles

Phase behavior of the Water/Synperonic^®^ 91/5/Isooctane system was investigated in order to define the compositions and temperatures at which microemulsions were formed, as detected by their transparency, low viscosity and optical isotropy (lack of birefringence) and high water content. The results obtained together with previous studies [27,31,32], allowed the selection of a specific composition with a water/surfactant/oil (W/S/O) weight ratio of 64.5/21.5/14 forming O/W microemulsions near room temperature. Copper (II) and cerium (III) 2-ethylhexanoate precursors were dissolved in isooctane forming the oil phase. Thus, the strategy was to vary the ratio of Cu (II) and Ce (III) 2-ethylhexanoate precursors dissolved in the oil phase, in order to have Cu/Ce molar ratios from 0/100 to 50/50. Then, the interval of temperature at which microemulsions with precursors were formed was investigated. The obtained O/W microemulsions were transparent and fluid, with bluish-green color due to the presence of copper precursor at various concentrations. Incorporation of the metal precursors promoted a shift in the temperature range at which O/W microemulsions were formed. The microemulsion without precursor was formed at a temperature range of 24–26 °C; whereas, when precursors where incorporated into the oil phase, the temperature needed for microemulsion formation was higher. Furthermore, as the content of copper precursor was increased, a higher temperature was needed for the formation of O/W microemulsions (Table 1).

In order to form the Cu/Ce mixed oxide nanoparticles, NaOH (2.5 M) was added, leading to the precipitation of Cu/Ce mixed oxide nanoparticles.

### 2.2. Characterization of Cu/Ce Oxide NPs

#### 2.2.1. High Resolution Transmission Electron Microscopy (HRTEM)

**Figure 1:**
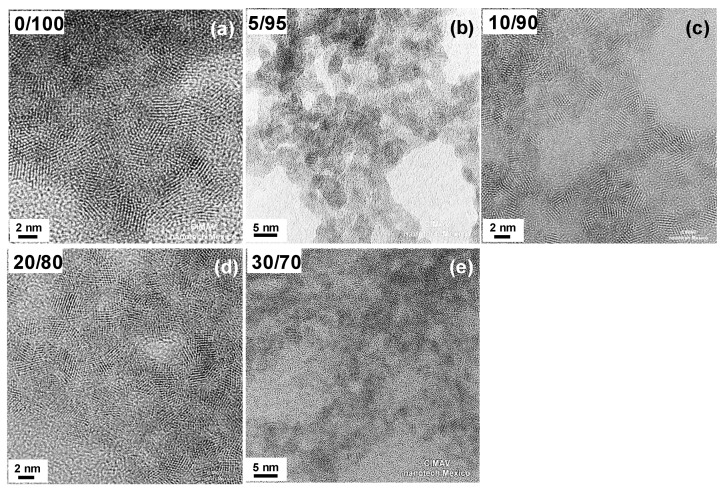
TEM micrographs of the synthesized nanomaterials (as obtained): (**a**) CeO_2_; (**b**) Cu_0.05_Ce_0.95_O_2-δ_; (**c**) Cu_0.10_Ce_0.90_O_2-δ_; (**d**) Cu_0.20_Ce_0.80_O_2-δ_; (**e**) Cu_0.30_Ce_0.70_O_2-δ_.

Assessment of crystallinity and particle size was carried out by HRTEM (JEOL-2200FS, Tokyo, Japan). Figure 1 shows high resolution TEM images of non-calcined (as prepared) mixed oxide nanoparticles with different Cu/Ce molar ratio (from 0/100 to 30/70). In all cases, the particles formed agglomerates of different sizes. These agglomerates are comprised of many nanoparticles with a diameter in the order of 2–3 nm. This homogeneous morphology and size was observed in samples containing Cu/Ce molar ratio from 0/100 to 30/70, as observed in Figure 1a–e, which are representative images of these samples. Crystalline features can be observed in these samples, indicating that the agglomerates were made up of small nanocrystals, and the size of the nanocrystals as well as the agglomeration decreased as the Cu content was increased (this can be seen in Figure 1 as well as in Figure S1, which shows a general low magnification view of all samples). Analysis of the Fast Fourier Transform (FFT) images of several crystals for samples with Cu/Ce molar ratio up to 20/80, indicated the presence of the {111} plane of cubic CeO_2_ (d-spacing of 0.3125 nm, ICDD PDF No. 98-001-1731; examples of analyzed crystals of all samples are shown in Supplementary Materials, insets to Figure S1 and in more detail Figure S2). For these samples, only small crystals corresponding to the CeO_2_ cubic fluorite phase were identified. For the sample with Cu/Ce molar ratio 30/70, the crystalline features were more disordered.

**Figure 2:**
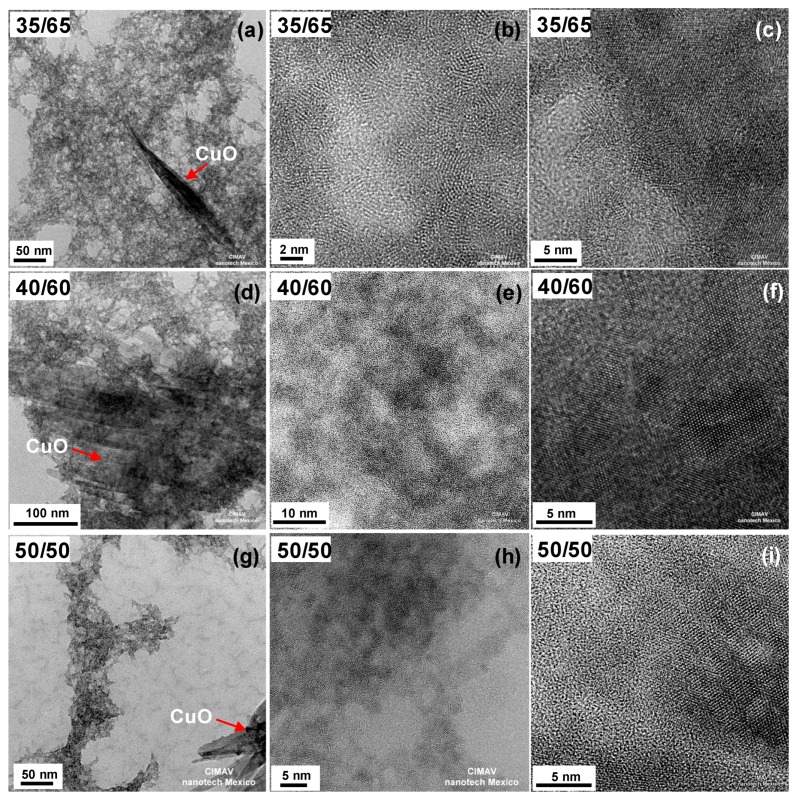
TEM micrographs of the synthesized nanomaterials (as obtained): Cu_0.35_Ce_0.65_O_2-δ_ (**a**) NPs and nanotape morphology; (**b**) NPs morphology and (**c**) nanotape morphology; Cu_0.40_Ce_0.60_O_2-δ_ (**d**) NPs and nanotape morphology; (**e**) NPs morphology and (**f**) nanotape morphology; Cu_0.50_Ce_0.50_O_2-δ_ (**g**) NPs and nanotape morphology; (**h**) NPs morphology and (**i**) nanotape morphology. Red arrows indicate CuO nanotapes (or nanotapes assemblies).

Figure 2a–i shows TEM micrographs for samples with Cu/Ce molar ratio 35/65, 40/60 and 50/50. For these samples, two morphologies were identified. At Cu/Ce molar ratio 35/65, a minor population of thin nanotapes of ~10–20 nm width in coexistence with the small nanoparticles was also found (Figure 2a–c). For samples with Cu/Ce molar ratio 40/60 and 50/50, the nanotapes were more abundant with a greater tendency to self-assemble (Figure 2d–i). These observations indicate that above Cu/Ce molar ratio 30/70, Cu atoms are no longer incorporated into the CeO_2_ matrix (or its concentration is too high to remain well-dispersed), promoting their growth into thin CuO nanotapes.

As the Cu/Ce molar ratio was increased above 30/70, the size of the nanocrystalline domains decreased for the small nanoparticles morphology (Figure 2b,e,h). In fact, for the samples with Cu/Ce molar ratio 40/60 and 50/50, the crystalline features of the mixed oxide nanoparticles were scarce, thus this component of the material was practically amorphous, as shown in Figure 2e,h. On the other hand, Figure 2c,f,i show representative high-resolution images of the thin nanotapes. FFT analysis of the nanotape in Figure 2c indicates the presence of planes {111}, {−112}, {020}, {022}, {220}, {311} and {−131} of monoclinic CuO (corresponding to d-spacings of 0.232 nm, 0.196 nm, 0.171 nm, 0.142 nm, 0.138 nm, 0.130 nm and 0.109 nm, respectively; ICDD PDF No. 98-000-6038). Similar results were obtained with the FFT analysis of the nanotapes in Figure 2f,i. It was observed that the continuity of the crystalline planes of the monoclinic CuO nanotapes has a long range. The dimensions of these particles was about 10–20 nm in width and several hundred nanometers in length. Similar results were obtained for nanotapes of the samples with Cu/Ce molar ratio 35/65 and 50/50. Thus, at Cu/Ce molar ratio ≥35/65 crystalline, monoclinic CuO nanotapes were formed under mild conditions, in addition to the mixed Cu/Ce oxide small nanoparticles.

**Figure 3:**
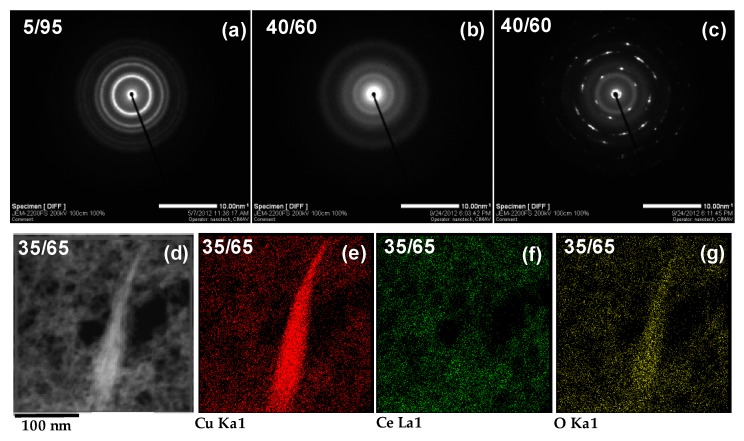
Electron diffraction patterns of the selected samples (as obtained): (**a**) Cu_0.05_Ce_0.95_O_2-δ_; (**b**) Cu_0.40_Ce_0.60_O_2-δ_ (corresponding to nanoparticle morphology); (**c**) Cu_0.50_Ce_0.50_O_2-δ_ (corresponding to nanotape morphology); EDX elemental mapping for the Cu_0.35_Ce_0.65_O_2-δ_ sample (as obtained): (**d**) bright field image; (**e**) Cu mapping; (**f**) Ce mapping; (**g**) O mapping.

Evolution of the phases was confirmed by acquiring selected area electron diffraction patterns (SAED). Examples of diffraction patterns of samples synthesized with Cu/Ce molar ratio 5/95 and 40/60 are shown in Figure 3a–c. The pattern in Figure 3a show the faint rings characteristic of polycrystalline materials for the sample with Cu/Ce molar ratio 5/95; similar results were obtained up to Cu/Ce molar ratio 30/70, although as the Cu/Ce molar ratio was increased, the rings became less defined, in agreement with the observations from HRTEM images. These rings were indexed by comparing with the corresponding powder diffraction file (PDF) from the International Centre for Diffraction Data (ICDD): face centered cubic Cerium Oxide (CeO_2_ ICDD PDF No. 98-001-1731). For the sample with Cu/Ce molar ratio 40/60, no defined rings were observed in the material with small nanoparticle morphology, as shown in Figure 3b, confirming the amorphous nature of these particles; similar SAED patterns were obtained for the nanoparticle morphology of samples with Cu/Ce molar ratios 35/65 and 50/50. In contrast, the diffraction spots shown in Figure 3c correspond to a nanotape crystal of the sample with Cu/Ce molar ratio 40/60, which were indexed as the diffraction planes {111}, {110}, {200}, {020}, {-311}, {022}, {113}, {400}, {−131}, and {220} corresponding to the monoclinic CuO phase (ICDD PDF No. 98-000-6038). Similar results were obtained for the nanotape morphology of samples with Cu/Ce molar ratio 35/65 and 50/50.

In order to analyze the chemical element distribution and clearly relate it with the different morphologies and phases found by HRTEM analysis for certain samples, Energy Dispersive X-ray Spectroscopy (EDX) elemental mapping of the sample containing Cu/Ce molar ratio 35/65 was carried out and the results are displayed in Figure 3d–g. This sample was deposited onto an Au TEM grid in order to avoid contribution from the conventional Cu grid. The elements detected were Cu, Ce, and O. A Cu signal was detected in both the nanoparticles and the nanotapes. A cerium signal was observed in the nanoparticles, whilst an oxygen signal was found homogeneously distributed in the entire sample. This information corroborates the aforementioned assignment of phases: in the small nanoparticles with cubic CeO_2_ structure, Cu and Ce are homogeneously distributed suggesting the formation of a mixed Cu-Ce oxide or an excellent dispersion of Cu onto CeO_2_, whilst the thin nanotapes are composed only by CuO.

#### 2.2.2. X-ray Diffraction

**Figure 4:**
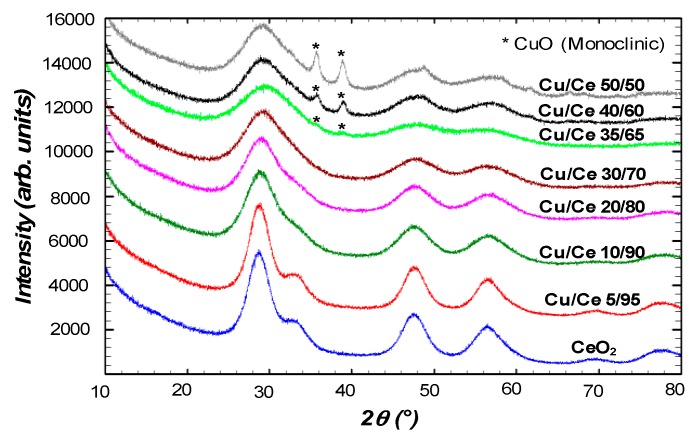
X-ray diffraction (XRD) patterns of samples (non-calcined) as a function of Cu/Ce molar ratio. The reflections marked as (*) are consistent with monoclinic CuO; the rest of the reflections are consistent with cubic fluorite-type ceria.

The overall crystallinity of the non-calcined (as obtained) samples was further studied by XRD and results are presented in Figure 4. Below Cu/Ce molar ratio 35/65, the crystal structure of mixed Cu/Ce oxide nanoparticles (Cu_*x*_Ce_1-*x*_O_2-__δ_) was identified with the characteristic reflections of the fluorite-type face-centered cubic ceria (CeO_2_) phase (ICDD PDF No. 98-001-1731). As the content of Cu increased, the characteristic reflections became wider, indicating lower crystallinity, in agreement with the results from HRTEM, which showed smaller and less defined crystalline domains. As shown in Table 2, the position of the main reflection was shifted from 28.71° (CeO_2_) to 29.35° (Cu/Ce molar ratio 35/65), which may suggest the incorporation of Cu into the CeO_2_ FCC lattice, since a shift of 2θ to larger values is an indication of smaller characteristic lattice dimensions, which is in agreement with the smaller size of Cu^2+^ as compared to Ce^3+^ or Ce^4+^ ions. However, due to the large broadening of the reflections of the as-obtained samples, this shift may be overestimated. On the other hand, for the sample with Cu/Ce molar ratio 35/65, additional reflections were detected and confirmed the presence of CuO monoclinic phase (ICDD PDF No. 98-000-6038). Thus, at Cu/Ce molar ratios 35/65, 40/60, and 50/50, samples were composed of a mixture of oxides with a main phase of Cu/Ce mixed oxide and an excess of monoclinic CuO phase. In addition, in agreement with HRTEM results at the compositions with Cu ≥35%, although the characteristic reflections for cubic ceria were observed, these reflections were very wide, indicating that crystallinity was rather poor. The crystallite size (*d*_XRD_) of the Cu/Ce mixed oxide nanoparticles was estimated using the Scherrer equation as a function of Cu/Ce molar ratio. The *d*_XRD_ and the corresponding 2θ for all samples are collected in Table 2.

**Figure 5:**
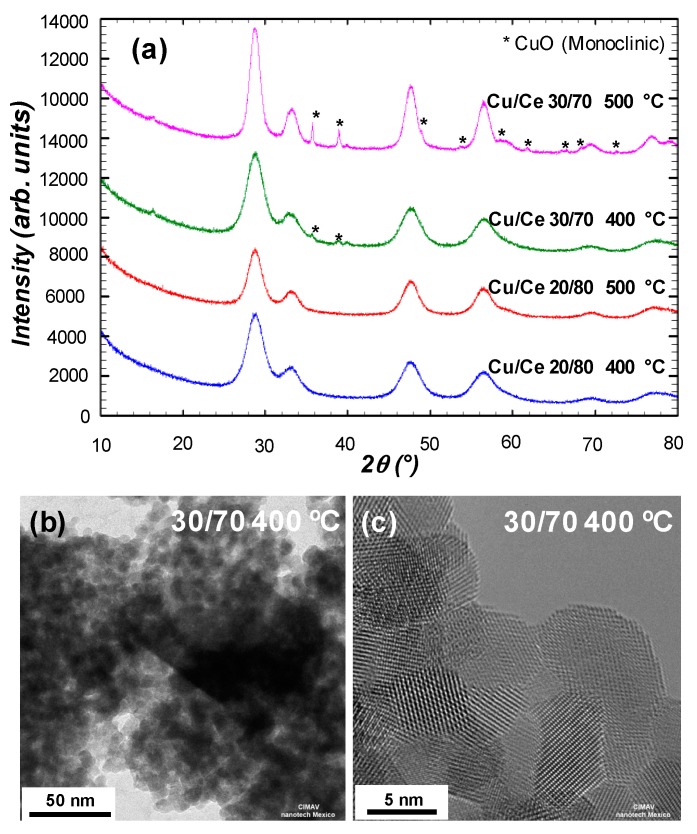
(**a**) XRD patterns of samples with Cu/Ce molar ratio 20/80 and 30/70, calcined at 400 °C and 500 °C. High-resolution transmission electron microscope (HRTEM) images of Cu/Ce 30/70 calcined at 500 °C; (**b**) showing a mixture of Cu/Ce mixed oxide nanoparticles and elongated CuO; and (**c**) atomic resolution of Cu/Ce mixed oxide nanoparticles.

Samples containing Cu/Ce molar ratios 20/80 and 30/70 were calcined at 400 °C and 500 °C so that the incorporation of Cu into the CeO_2_ crystal lattice could be investigated with consistence. The characteristic reflections of the fluorite-type cubic crystal structure were still observed for both calcined materials except that their reflections were much more defined than before calcination (Figure 5a). This observation can be explained by the increase of crystallite size as approximated by the Scherrer equation and values are presented in Table 3. The main reflection presented a much lower shift in the calcined samples, remaining close to 28.7° (28.74°–28.77°), in contrast to the corresponding non-calcined samples (28.93°–29.12°). The nanomaterial containing Cu/Ce molar ratio 20/80 presented no additional reflections after calcination. However, the sample with Cu/Ce molar ratio 30/70 presented additional reflections, characteristic of monoclinic CuO. HRTEM images in Figure 5b,c confirmed the formation of the CuO phase in the form of elongated structures upon calcination as well as the increased crystallinity of the Cu/Ce mixed oxide nanoparticles.

#### 2.2.3. Raman Spectroscopy

**Figure 6:**
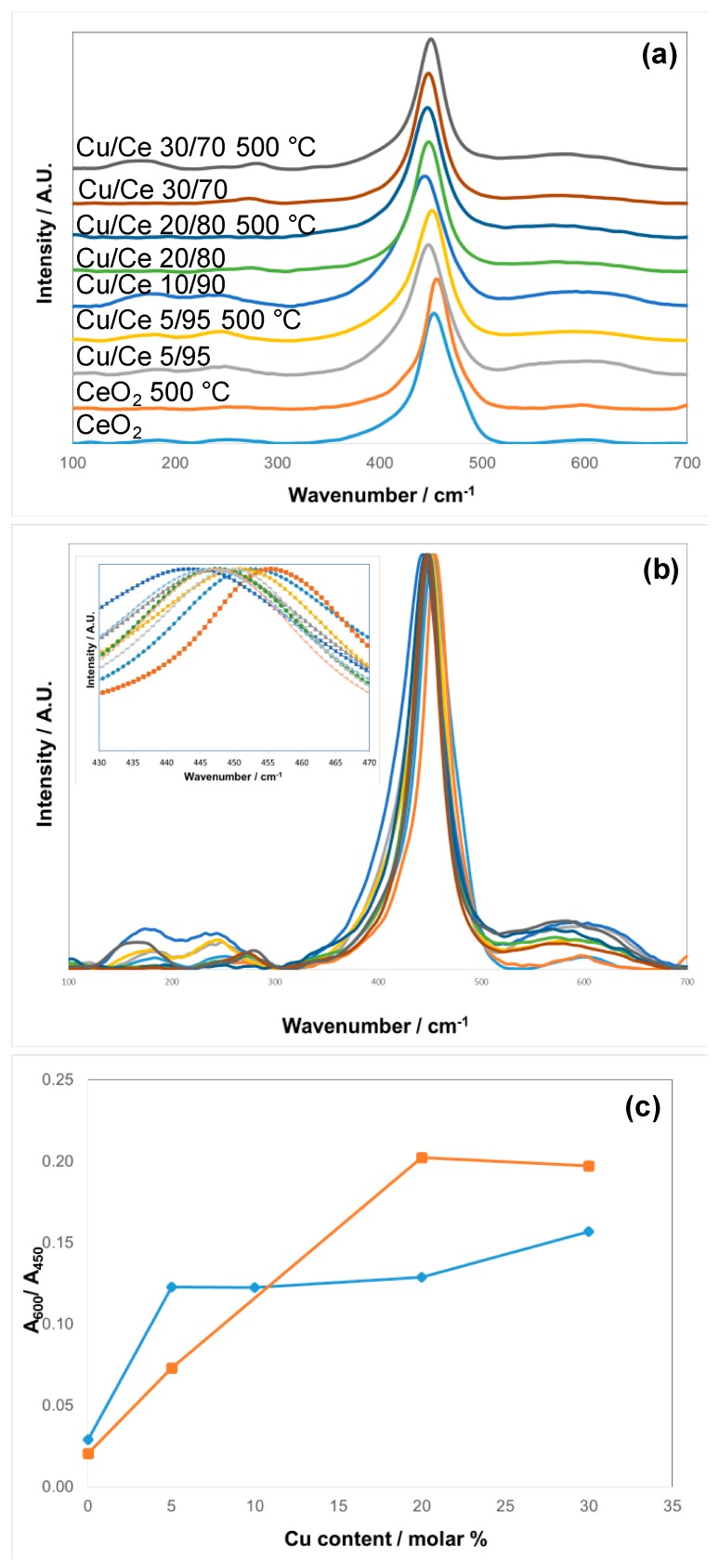
(**a**) Raman spectra of CeO_2_ and Cu/Ce mixed oxides (as-obtained and calcined at 500 °C); (**b**) Same data without offset for comparison of relative intensity of the different bands; inset: zoom into the F_2g_ Raman active mode; (**c**) A_600_/A_450_ for assessment of relative oxygen vacancies as a function of Cu content: (♦) as-obtained and (■) calcined samples. Symbols (inset (**b**)): (■) CeO_2_; (♦) CeO_2_ 500 °C; (▲) Cu/Ce 5/95; (×) Cu/Ce 5/95 500 °C; (*) Cu/Ce 10/90; (●) Cu/Ce 20/80; (**+**) Cu/Ce 20/80 500 °C; (**-**) Cu/Ce 30/70; (**—**) Cu/Ce 30/70 500 °C.

Raman Spectroscopy was used for further investigation of the structure of selected non-calcined (as prepared) and calcined samples. The main feature of such CeO_2_ based materials is the F_2g_ Raman active mode of the CeO_2_ fluorite phase, which for bulk CeO_2_ it is located at 465 cm^−1^ [33], whereas for nanosized CeO_2_ materials it has been reported in the range of 460–466 cm^−1^ [34]. The Raman spectra for these fluorite-type oxide structures are dominated by oxygen lattice vibrations and are sensitive to crystalline symmetry [35]. Figure 6a,b shows the Raman spectra; for comparison purposes, the intensities have been normalized. The samples display the characteristic and relatively intense F_2g_ band, centered at 452 and 455 cm^−1^ for CeO_2_ as-obtained and calcined at 500 °C (CeO_2_ and CeO_2_ 500 °C); 447 and 451 cm^−1^ for Cu/Ce 5/95 and Cu/Ce 5/95 500 °C; 443 cm^−1^ for Cu/Ce 10/90; 446 and 447 cm^−1^ for Cu/Ce 20/80 and Cu/Ce 20/80 500 °C; and 447 and 449 cm^−1^ for Cu/Ce 30/70 and Cu/Ce 30/70 500 °C (see shift in the inset of Figure 6b). The other important feature of the Raman spectra is the band extending from 500 to 650 cm^−1^ (Figure 6c); this band has been assigned to oxygen vacancies [36]. Other bands were observed between 100 and 300 cm^−1^.

### 2.3. Magnetic Properties

**Figure 7:**
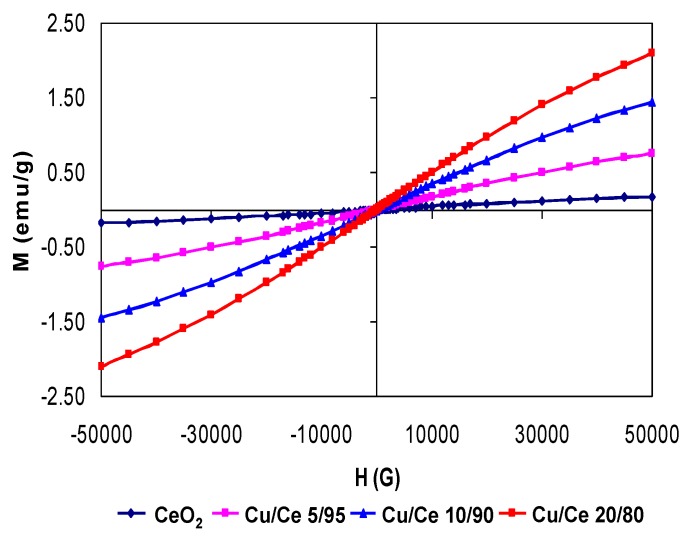
Magnetization as a function of the magnetic field at 5 K of nanoparticles (CeO_2_, Cu_0.05_Ce_0.95_O_2-δ_ (Cu/Ce 5/95), Cu_0.10_Ce_0.90_O_2-δ_ (Cu/Ce 10/90), Cu_0.20_Ce_0.80_O_2-δ_ (Cu/Ce 20/80).

Assessment of the magnetic properties of selected samples was carried out at 5 K (−268.15 °C) in order to find out the influence of incorporating Cu into CeO_2_ (Figure 7). At 300 K the magnetic response of CeO_2_ nanoparticles was very small upon applying a magnetic field up to 50 kG whereas at 5 K, a certain magnetic response was noticed. This magnetic behavior may be explained by the surface defects of CeO_2_ nanoparticles. A comparison of the magnetic properties of two Cu/Ce mixed oxides was also carried out (Figure 7). At a higher amount of Cu in the CeO_2_ lattice, a higher magnetization response is achieved. Indeed, for pure CeO_2_ the maximum magnetization response at *H* = 5 kG is ~0.25 emu/g whereas for Cu/Ce 20/80, the maximum magnetization response is ~2.1 emu/g. It should be noted that by applying a magnetic field up to 50 kG the magnetization saturation was not achieved which means that all the spins were not yet aligned at lower magnetic fields. Therefore, by doping CeO_2_ lattice with Cu, the magnetic properties could be tuned from diamagnetic to paramagnetic behavior since no hysteresis could be detected.

### 2.4. Optical Properties and Photocatalytic Degradation of Indigo Carmine

**Figure 8:**
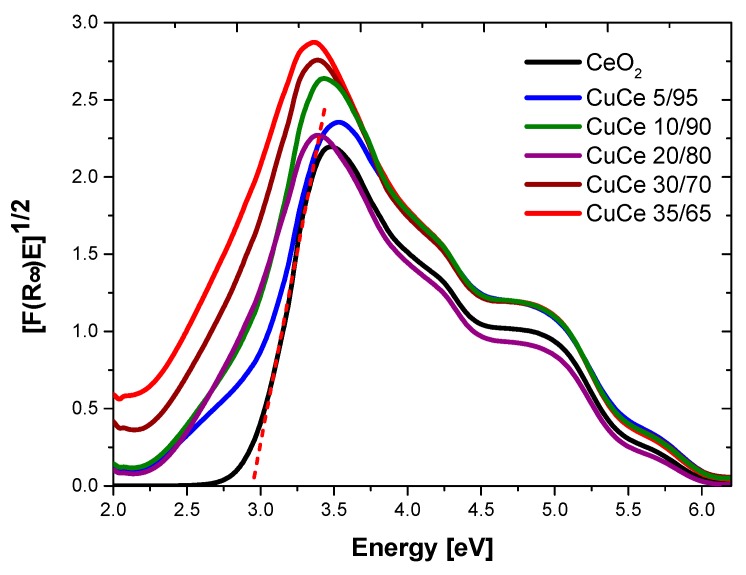
Kubelka Munk absorption spectra of non-calcined nanomaterials at different Cu/Ce molar ratio.

The bandgap energy (*E_g_*) of Cu/Ce samples was calculated by Kubelka-Munk function [37] as shown in Figure 8. It is clearly observed that all samples exhibited absorption in the visible light interval, and the *E*_g_ values are lower than 3.0 eV, calculated *E*_g_ are shown in Table 4. The *E_g_* value of CeO_2_ nanoparticles was 2.91 eV, whereas samples containing different Cu/Ce molar ratio resulted in *E_g_* values in the order of 2.85–2.27 eV. 

**Figure 9:**
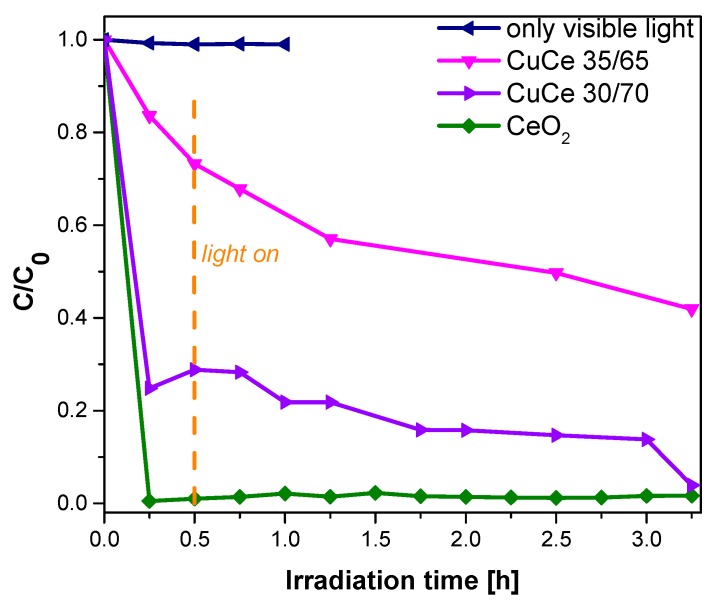
C/C_0_ *versus* irradiation time for the degradation of indigo carmine using CeO_2_, Cu/Ce (30/70 and 35/65)) under visible light irradiation.

The photodegradation of Indigo Carmine using Cu/Ce (30/70 and 35/65) oxides as photocatalyst was explored under visible light. Figure 9 shows the evolution of C/C_0_ including the curve obtained from the photolysis and the use of the CeO_2_ sample as photocatalyst for comparison purposes. Accordingly, it is observed that CeO_2_ immediately adsorbs the dye completely, whereas samples containing copper show less dye adsorption, increasing the photodegradation of the dye. In this case, the use of Cu/Ce samples as photocatalyst allow the photodegradation of about 60% of the initial dye concentration after 3 h for sample Cu/Ce 35/65; whereas sample Cu/Ce 30/70 reached almost 100% removal in the same time, but this sample is still experiencing rather strong dye adsorption. In conclusion, the increment of Cu in the Cu/Ce samples diminishes the absorption and enhances the photodegradation of the Indigo Carmine.

## 3. Discussion

### 3.1. Microemulsion Formation and Particle Synthesis

The slight variation of temperature at which microemulsions were formed when the Cu/Ce ratio was increased may be explained by a different interfacial activity of the Cu (II) and Ce (III) 2-ethylhexanoate precursors, affecting the hydrophilic-lipophilic balance of the surfactant placed at the O/W interface to a different extent. This suggestion is in agreement with the findings of Oliveira *et al.* In their study, spectroscopic analysis was used to demonstrate that a similar precursor, cobalt (II) 2-ethylhexanoate doped in water/AOT/heptane W/O microemulsions, resides at the O/W interface [38]. In addition, our research group investigated the phase behavior of a water/surfactant/oil system very similar to the system used in the present study, in which cerium (III) 2-ethylhexanoate precursor was incorporated in the oil phase [31]. It was demonstrated that the presence of this precursor resulted in the modification of the O/W microemulsion region in pseudo-ternary phase diagrams. The fact that higher temperature was needed for microemulsion formation as the concentration of Copper (II) precursor was increased may be explained as follows. Ce (III) precursor has three units of 2-ethylhexanoate complexing agent, whereas Cu (II) precursor has two units. Thus, it can be assumed that the steric effect is reduced with Cu (II) precursor leading to better packing at the oil/water interface (in a similar manner as Cobalt (II) 2-ethylhexanoate), as compared to the bulkier Ce (III) precursor. Thus, the hydrophilic-lipophilic balance is affected to a greater extent when the concentration of Cu (II) precursor is increased.

In any case, it was possible to carry out the reactions close to room temperature (27–30 °C). A simple process approach such as the one presented in this investigation can be applied to further studies such as scaling up. Furthermore, the nonionic surfactant used is a low cost technical grade material which is biodegradable, and given that its solubility depends greatly on temperature further studies may permit its reuse.

Regarding the formation of Cu/Ce mixed oxide nanoparticles in the O/W microemulsions, due to the fact that the metal-organic precursors are dissolved in the oil phase, and the precipitating agent is soluble in the aqueous phase, the reaction should start at the oil/water interface, forming the first nuclei. Considering the small size of microemulsion droplets (in the order of 5 nm, as characterized by dynamic light scattering), and the huge oil/water interfacial area, there is a great level of confinement for the reaction to occur, resulting in the formation of small nanoparticles with a great level of interaction at the nanoscale level between the two precursors, leading to highly homogeneous materials up to Cu/Ce molar ratio 30/70, as demonstrated by the different characterization techniques.

### 3.2. Characterization of Cu/Ce Oxide Nanoparticles

HRTEM studies showed that rather small NPs were obtained: about 2–3 nm for non-calcined samples and about 5–7 nm for calcined samples. Homogeneity of materials below Cu/Ce molar ratio 30/70 as shown by HRTEM, EDX, and XRD spectra may be attributed to the fact that the precursors are confined inside the microemulsion droplets at the nanometer level, leading to an increased synergy between the oxides. Furthermore, the increase of Cu/Ce molar ratio promoted a decrease of crystallite size, which may suggest that either enrichment of Cu^2+^ on the surface of CeO_2_ or Cu^2+^ incorporated into the CeO_2_ lattice, inhibited the crystal growth of ceria. The ionic radius of Cu^2+^ (0.072 nm) is smaller than Ce^4+^ (0.097 nm) and Ce^3+^ (0.1143 nm), thus, an increasing amount of Cu into the CeO_2_ lattice can lead to a reduction of its cell size [25]. However, incorporation of Cu into the cubic CeO_2_ lattice would also imply a shift in the position of the main reflection to higher 2θ values. Indeed, for non-calcined samples there appears to be a shift in the position of the main reflection to higher values; in contrast, for the calcined samples, this shift is rather small with respect to CeO_2_ (2θ = 28.7° for CeO_2_ *versus* 28.74°–28.77° for calcined samples). Thus, the apparent larger shift in the non-calcined samples is more likely to be attributed to the large broadening of the reflections caused by the small size of the domains, which prevents an accurate positioning of the maximum. Furthermore, the fact that the agglomeration of the nanoparticles decreases as the Cu content increases (Figure 1), supports a change in the surface free energy of the nanoparticles, which could be ascribed to enrichment of Cu^2+^ on the surface, e.g., a good dispersion of Cu^2+^ ions onto CeO_2_ [7,39]. Although, partial substitution of Ce^4+^/Ce^3+^ by Cu^2+^ in the ceria lattice cannot be ruled out.

Even though in the Cu/Ce 30/70 non-calcined sample only small particles were observed by HRTEM and CuO phase was not detected by XRD, after calcination at 400 and 500 °C some excess monoclinic CuO phase was indeed detected by both techniques (Figure 5). Thus, it is inferred that CuO was formed initially as a very well dispersed secondary amorphous phase. Calcination caused phase segregation and crystallization of monoclinic CuO for this sample, whereas for the sample with Cu/Ce molar ratio 20/80 no phase segregation of monoclinic CuO was observed even after calcination at 500 °C. On the contrary, for the samples with Cu/Ce molar ratio 35/65, 40/60, and 50/50, Cu^2+^ ions concentration was too high to remain completely well dispersed, and it partially segregated as CuO in the as-obtained samples. The fact that this segregated CuO was monoclinic even without calcination, evidences the strong interaction between Cu^2+^ ions and ceria in the mixed oxide synthesized by this method, that prevents its segregation as monoclinic CuO up to Cu/Ce molar ratio 20/80 even after thermal treatment.

The Raman spectra indicated a slight shift towards lower wavenumber for the F_2g_ Raman active mode of the CeO_2_ fluorite phase as the concentration of Cu was increased (Figure 6b), illustrating that the CeO_2_ fluorite-type lattice was distorted with the incorporation of Cu^2+^ ions into CeO_2_. On the other hand, the widths of the F_2g_ bands are similar, due to very small and similar crystallite size obtained in all cases. For CeO_2_ in the present study, the characteristic and relatively intense F_2g_ band, was centered at 452 and 455 cm^−1^ (non-calcined and calcined at 500 °C, respectively). This is in agreement with the work by Spanier *et al.*, who reported a Raman shift of 458 cm^−1^ for CeO_2_ nanoparticles of 6.1 nm, and a general shift towards lower wavenumber as the particle size of CeO_2_ is decreased [40]. Thus, it is expected that smaller particles have a Raman shift towards lower wavenumber. According to Wang *et al.*, the F_2g_ band of a sample with 20 molar% of Cu prepared using W/O microemulsions was centered at 457 cm^−1^, as compared to CeO_2_ synthesized by the same method which was centered at 463.5 cm^−1^ [41]. Thus, a shift of about 6 cm^−1^ was observed when the Cu/Ce molar ratio was 20/80. In the present study, Cu/Ce with molar ratio 20/80 was shifted to 446 and 447 cm^−1^ (as-obtained and calcined at 500 °C, respectively). Thus, the shift was in the order of 6–8 cm^−1^. A similar shift was obtained for the sample with Cu/Ce molar ratio 30/70. Therefore, the shift is comparable to the shift obtained in the investigation reported by Wang *et al.* [41], who interpreted this outcome to the incorporation of Cu into the CeO_2_ cubic lattice. Although, as mentioned previously, this should be accompanied by Cu^2+^ surface enrichment, since the XRD reflections presented a rather small shift [42]. In any case, given the small size of the Cu/Ce mixed oxide nanoparticles, a very large percentage of the atoms must reside at these surface/subsurface locations.

As mentioned previously, the band at 500–650 cm^−1^ can be assigned to oxygen vacancies [36]. For CeO_2_, this is in agreement with the presence of an important amount of Ce^3+^, as already reported for CeO_2_ synthesized by the O/W microemulsion method [43]. On the other hand, the increase in the intensity of this band in the Cu/Ce mixed oxides may be attributed to the incorporation of Cu^2+^ ions into the ceria lattice, which must be accompanied by the generation of oxygen vacancies in order to keep the charge balance. A way to assess the relative oxygen vacancies in a comparative manner is to express the ratio of the area of the vacancies band (A_600_) with respect to the area of the F_2g_ band (A_450_) [44]. For the samples calcined at 500 °C, there was a steady increase in the A_600_/A_450_ ratio up to Cu/Ce molar ratio 20/80, after which it leveled off (Figure 6c). This could be explained by the fact that for the Cu/Ce 30/70 sample calcined at 500 °C an important amount of CuO had phase-separated, leaving the Cu/Ce mixed oxide with a composition similar to the Cu/Ce 20/80 sample. On the other hand, the as-obtained samples presented a rapid increase at Cu/Ce 5/95 as compared to CeO_2_, with little increase above this Cu/Ce molar ratio. Thus, calcination induced an increase of oxygen vacancies as a function of Cu concentration, as compared to the as-obtained samples; this can be ascribed to the increased crystallinity, allowing for a better incorporation of Cu^2+^ into the CeO_2_ lattice.

Between 100–300 cm^−1^ there are some other bands which have not been usually reported in these Cu/Ce mixed oxides. Samples Cu/Ce 30/70 (as-obtained and calcined at 500 °C ) present a small band centered at 272 and 280 cm^−1^ (respectively) which could be attributed to the one-phonon A_1g_ mode of CuO [45]. This is in agreement with the results from XRD for the calcined sample; for the as-obtained sample CuO was not detected, so probably it was very well dispersed and/or in a concentration below the limits of the technique. The other small bands at around 180 and 250 cm^−1^, are probably also related to the oxygen vacancies, since these are also present in the CeO_2_ samples, and they follow the trends of the bands at 500–650 cm^−1^ [44].

A systematic study on the preparation of Cu/Ce oxide nanoparticles at various Cu/Ce molar ratios using the flame method was previously reported by Pati *et al.* [46]. They found that below 40/60 Cu/Ce molar ratio, crystalline CuO could not be detected by XRD, however it was demonstrated by HRTEM that a core-shell structure was obtained, in which fluorite-type CeO_2_ formed the core and amorphous CuO formed the shell. Thus, even though the temperature of synthesis reached 1500 °C in the flame method, a mixed Cu/Ce oxide phase was not obtained and the CuO phase was amorphous. In the present study, no clear evidence for a core-shell configuration was found by HRTEM, however the surface may be enriched with a thin layer of Cu^2+^ ions. For the higher concentrations of Cu (Cu/Ce molar ratios 35/65, 40/60, and 50/50) HRTEM showed amorphous zones. Nonetheless, in our investigation it has been shown that if the Cu/Ce ratio is high enough (35/65 and above), monoclinic CuO is formed, even at mild reaction conditions. Thus, the amorphous zones for samples with Cu/Ce molar ratios 35/65, 40/60, and 50/50 are more likely to be formed by the mixed Cu/Ce oxide.

It is worth noting that various configurations of Cu/Ce oxide materials have been obtained depending on the preparation method, such as the core-CeO_2_/shell-CuO mentioned above, as well as nanoparticles or films of mixed Cu/Ce oxide in which Cu is either very well dispersed or incorporated into the CeO_2_ lattice [39,41,47,48,49,50,51]. However, the limit of Cu content that can be incorporated without forming an excess separate CuO phase depends strongly on the preparation method and reaction conditions. With the impregnation method, it was reported by Yang *et al.* that excess monoclinic CuO phase can be detected by XRD at Cu/Ce molar ratio 5/95 [47]. In a similar investigation by Zheng *et al.*, monoclinic CuO phase was detected from 10/90 Cu/Ce molar ratio by the impregnation method [48], although Tian *et al.* reported the incorporation of Cu up to a 15/85 Cu/Ce molar ratio using the same preparation method [49]. Using the urea precipitation-gelation method, it was possible to incorporate Cu up to a 15/85 Cu/Ce molar ratio, according to Kobayashi *et al.* [39]. Slusser *et al.* reported the formation of epitaxial films by the pulsed laser deposition technique, in which Cu was incorporated into the CeO_2_ lattice up to a 15/85 Cu/Ce molar ratio; higher Cu content was not reported [52]. By a modified citrate sol-gel method, monoclinic CuO phase was detected by XRD at 20/80 Cu/Ce molar ratio [50]. Fotopoulus *et al.* reported the synthesis of mixed Cu/Ce oxide in which Cu was incorporated into the CeO_2_ lattice up to a 23/77 Cu/Ce molar ratio, or it was very well dispersed; the synthesis was carried out by a solvothermal method using oleylamine/TOPO mixtures at 350 °C [51]. Wang *et al.* reported that by the reverse microemulsion reaction method, only cubic fluorite-type ceria structure was observed for samples with up to 20/80 Cu/Ce molar ratio [41].

The experimental advantages of preparing mixed Cu/Ce oxide nanoparticles by the O/W microemulsion reaction method are the use of mild conditions (room temperature), lower concentration of solvents as compared to the solvothermal or W/O microemulsion methods, and the use of simple and inexpensive equipment. In addition, the homogeneity and confinement of the molecular mixture of Cu/Ce precursors inside the microemulsion droplets during synthesis, and the narrow size distribution (low polydispersity) obtained, may promote a stronger synergistic effect between copper and ceria leading to an improved performance in catalysis such as in the Water-Gas Shift reaction and other catalytic processes [53].

### 3.3. Magnetic Properties

It was reported recently that certain transition metal-doped rare earth oxides such as CeO_2_ led to room temperature ferromagnetism [54,55,56]. Tiwari *et al.* reported that incorporation of cobalt into the CeO_2_ lattice resulted in room temperature ferromagnetism and high Curie Temperature [54]. Ferromagnetic behavior was also reported in Ni- and Fe-doped CeO_2_ [55,56]. A significant debate exists about whether the observed properties are an intrinsic property of the material or an extrinsic property due to the preparation method, presence of impurities and so on. This controversy still remains intense since all the materials prepared as transition metal-doped CeO_2_ are magnetic even though a very small amount of the dopant is used [54,55,56]. In the present investigation, it was clearly observed that by incorporating non-magnetic Cu^2+^ ions into CeO_2_, the magnetization response was enhanced.

Slusser *et al.* [52] found unexpected ferromagnetic behavior in Cu-doped CeO_2_ films prepared by a pulse laser deposition technique at room temperature. This magnetic behavior could not be detected for the same materials prepared in the O/W microemulsion method. This difference in magnetic behavior may be related to the nanoparticle size difference or cation partition in the sample. Indeed, in the present study the nanoparticle size was ~2–3 nm whereas in the study by Slusser *et al.*, although no size detail was given, based on the resolution of XRD reflections, it can be inferred that the characteristic crystallite size was much larger.

### 3.4. Optical Properties and Photocatalytic Activity

In the literature, various values of *E*_g_ have been reported for CeO_2_ nanostructures, depending on the preparation method. Ho *et al.* [57] reported *E*_g_ values from 3.36 to 3.62 for mesoporous CeO_2_ nanostructures with different morpohologies (rod shape and spindle-like), synthesized by polyol method. In contrast, by the precipitation method higher *E*_g_ (3.56 to 3.71 eV) were reported for CeO_2_ [58]. Masui *et al.* reported slightly lower *E*_g_ values (3.38 and 3.44 eV) when CeO_2_ NPs with sizes between 2.6 and 3.4 nm were prepared in reverse micelles [59]. It was reported by Patsalas *et al.* that the the red shifting in the band gap of CeO_2_ is due to an increase in the concentration of Ce^3+^ on the grain boundaries; the reduction of the band gap increases with increasing Ce^3+^ concentration and its corresponding increase in oxygen vacancies [60]. Thus, the relatively low *E*_g_ value obtained for CeO_2_ in the present study of 2.9 eV could be related to an important contribution from oxygen vacancies (as indicated from the Raman results), which is accompanied by a high concentration of Ce^3+^ as previously reported for CeO_2_ obtained in O/W microemulsions [43].

On the other hand, reports on the *E*_g_ values of Cu/Ce oxides are rare. In our study, it was shown by XRD and HRTEM techniques that the obtained Cu/Ce mixed oxide nanoparticles had a slightly smaller size as the concentration of Cu was increased. According to the study by Tsunekawa *et al.*, the *E*_g_ values of CeO_2_ nanoparticles should increase as the particle size is decreased, due to the electron confinement effect [61]. However, in this investigation, the incorporation of Cu into the cubic CeO_2_ lattice promoted a general decrease of *E*_g_ of Cu/Ce mixed oxide nanoparticles compared to CeO_2_ nanoparticles. Slusser *et al.* reported that the *E*_g_ values decreased as the concentration of Cu was increased for Cu/Ce nanostructured films [52]. However, the values in that study were 3.45, 3.43, and 3.39 eV for samples containing 0, 3 and 15 atomic% of Cu. The synthesis of CeO_2_ and Cu/Ce mixed oxide NPs using Oil-in-Water microemulsions as confined reaction media led to NPs possessing even lower *E*_g_ values which may be of high interest in optoelectronics, photovoltaic, and photocatalytic applications, since *E*_g_ values lower than 3 eV implies that the material may be photoactive under visible light irradiation.

The photocatalytic activity was confirmed in this investigation for the photodegradation of Indigo Carmine, which reached 60% after 3 h of irradiation with visible light for sample Cu/Ce (35/65) and almost 100% of photodegradation was reached when sample Cu/Ce (30/70) was used as photocatalyst. However, in this last case the dye is at least partially adsorbed by the sample due to the high amount of Ce. On the other hand, Indigo Carmine degradation is commonly due to the formation of several intermediates as reported in other works; it means that the formation of intermediates and the degradation of the chromophore group is a common step in the photodegradation process of this dye [62]. Therefore, in our case, the degradation of Indigo Carmine by using Cu/Ce with molar ratios 30/70 and 35/65 could be due to the sulphate ion separation and bonds breaking and arranging to form compounds with nitro groups and finally obtain carboxylic acids [62]. In any case, it was demonstrated that both CeO_2_ and the mixed Cu/Ce oxides synthesized in O/W microemulsions, can be used for the remediation of water to remove contaminants such as certain dyes, either by photocatalytic degradation or by adsorption, or both.

It is worth mentioning that a previous report from Torres-Martinez on the photodegradation of Indigo Carmine using Sm_2_FeTaO_7_ photocatalyst under solar light resulted in better performance when using CuO as cocatalyst, which was ascribed to CuO acting as an electron trap, decreasing electron-hole pair recombination rates [63]. This suggests a double advantage of having a well dispersed CuO phase as in the Cu/Ce oxide system reported in this investigation, or even an excess monoclinic CuO phase, for improving the photocatalytic performance of CeO_2_. On one hand, the red shift of the *E*_g_ value permits its photocatalytic activation under visible or solar light radiation, and on the other hand, the presence of CuO as cocatalyst acts as an electron trap.

## 4. Materials and Methods

### 4.1. Materials

Synperonic^®^ 91/5 (Polyoxyethylene (5) C9-C11 alcohol, HLB = 12, Cloud point T = 36 °C) was kindly obtained from Croda. Cerium (III) 2-ethylhexanoate (Ce-2EH, solid) was purchased from Sigma Aldrich, Copper (II) 2-ethylhexanoate (Cu-2EH, *ca* 98%) was purchased from Alfa Aesar. Isooctane (Suprasolv, for gas chromatography, 99% min) was from Honeywell Burdick & Jackson. Isopropanol (99.5%) and sodium hydroxide (NaOH, pellets, 98.4%) were purchased from J.T. Baker. Ethanol (99%) was purchased from CTR Scientific. Deionized water with a resistivity of 18.2 MΩ cm^−1^ was obtained from a Millipore system.

### 4.2. Preparation of NPs by the O/W Microemulsion Reaction Method

Microemulsions are well known as thermodynamic stable systems containing water, oil and surfactant leading to a transparent and fluid isotropic solution. The organometallic precursors (Ce-2EH and Cu-2EH were dissolved in isooctane composing the oil phase (which had a total metal concentration of 0.105 mol per kg of solution). Then, deionized water, Synperonic^®^ 91/5 and the oil phase (containing the precursors) were mixed. The composition of the microemulsion selected was: 64.5 wt % water, 21.5 wt % Synperonic^®^ 91/5, and 14 wt % oil. The mixture was magnetically stirred at the appropriate microemulsion temperature (Table 1) until a homogeneous, transparent and fluid isotropic single phase was obtained. In order to form the mixed oxide nanoparticles, an aqueous solution of NaOH (2.5 M) was added under vigorous stirring until pH 12 was reached, whilst the microemulsion temperature was kept constant. The reaction mixture was kept overnight at constant stirring, followed by centrifugation. The nanoparticles were washed with ethanol and water (*v*/*v* = 1/1) until a neutral pH was reached in the washing liquors. The solids were dried at 70 °C over 2 days, and ground using an agate pestle and mortar. The synthesized materials were CeO_2_ and Cu*_x_*Ce_1-*x*_O_2-*δ*_ (*x* = 0.05, 0.10, 0.20, 0.30, 0.35, 0.40, 0.50). Some materials were calcined with a temperature ramp of 5 °C/min and a dwelling time of 2 h; the calcination temperatures were 400 °C or 500 °C.

### 4.3. Characterization of NPs

Particle size and morphology were investigated using a High-Resolution Transmission Electron Microscope (HRTEM) JEOL JEM-2200FS, operated at 200 kV, and point resolution of 0.1 nm in TEM mode. Preparation of TEM grids was carried out by mixing a small amount of powder with isopropanol (2 mL) followed by sonication, and depositing a drop of this dispersion onto a formvar/carbon copper grid. The crystal structure was analyzed by X-ray diffraction (XRD (PANalytical, Almelo, The Netherlands)) using a PANalytical Empyrean diffractometer with CuK_α_ radiation in continuous scan mode from 10° to 80° of 2θ with 0.001 step size. Fullprof Suite software (2007, Grenoble, France) was used for data treatment. The crystallite size was estimated by X-ray Diffraction with the Scherrer equation using the broadening of the reflection with the highest intensity (2θ = 28.7°). Raman spectroscopy was carried out using a micro-Raman LabRAM HR Evolution from Horiba (Kyoto, Japan), coupled to an Olympus BX-4 microscope. The wavelength used to excite the sample was 632.8 nm, which was provided with a He-Ne laser, the power was kept at 17 mW, the resolution was 1 cm^−1^ and the diffraction grating was 600 L/mm; all measurements were performed at room temperature. The power of the laser was varied between 5% and 10%, depending on the sample.

### 4.4. Magnetic Properties, Optical Properties and Photocatalytic Performance

The magnetic properties of the obtained nanomaterials were assessed using superconducting quantum interface device (SQUID) magnetometer Quantum Design MPMS XL. The energy band gap (*E*_g_) of the powders was determined by the Kubelka–Munk function using a UV–vis spectrophotometer (Lambda 35 Perkin Elmer Corporation) coupled with an integrating sphere. The photocatalytic degradation of Indigo Carmine was carry out using 200 mL of a 20 ppm solution of the dye; 0.2 g of the photocatalyst were used for each experiment. Irradiation was carried out with a visible light lamp (Xenon lamp HID, 3200 Lumen, 6000 K Onof, Guangzhou, China). The development of the photocatalytic reaction was conducted by UV-vis analysis using a Lambda 35 Perkin Elmer Corporation spectrophotometer; samples were taken every 15 min during 180 min. Before turning on the Xenon lamp, 30 min in the dark were allowed for adsorption equilibria. The photolysis experiment was carried out in the same way, except that no photocatalyst was added.

## 5. Conclusions

Mixed Cu/Ce oxide nanoparticles (below Cu/Ce molar ratio 30/70) and mixtures of oxides (above this content) were prepared using the oil-in-water microemulsion reaction method under mild conditions. Nanoparticle size of mixed Cu/Ce oxide was in the order of 2–3 nm with a low polydispersity. However, calcination above 400 °C showed clearly the presence of a minor CuO phase for samples with Cu content equal or larger than 30%, evidencing the formation of mixed Cu/Ce oxide as a major phase and a minor phase of CuO nanotapes with a monoclinic crystal structure. Evidence from characterization suggests a contribution from Cu^2+^ surface enrichment, ascribed to the lack of a significant shift in the XRD reflections and lower agglomeration in the mixed oxide; however, the shift in the signal corresponding to the F_2g_ Raman active mode of the CeO_2_ fluorite phase and the increase of the Raman band ascribed to oxygen vacancies, suggest at least partial incorporation of Cu^2+^ into the CeO_2_ lattice. Nanoparticle characteristics were modulated by the variation of Cu/Ce molar ratio in the O/W microemulsion system. By incorporating Cu into CeO_2_, the magnetic properties could be tuned from diamagnetic to paramagnetic behavior. Moreover, the band gap (*E*_g_) values of these nanomaterials were tuned and decreased as a function of Cu/Ce ratio. These results are also consistent with at least partial incorporation of Cu^2+^ into the CeO_2_ lattice. CeO_2_ NPs prepared in O/W microemulsions possessed a lower *E*_g_ (2.9 eV) than those prepared using other methods as reported in literature. The obtained *E*_g_ values imply that both CeO_2_ and Cu/Ce oxides may be photoactive under visible light. Indeed, it was shown for the first time that Cu/Ce oxide nanoparticles are active as photocatalyst for the degradation of Indigo Carmine dye using visible light irradiation. These properties may promote their use as promising materials in photocatalytic, optoelectronic, photovoltaic, and fuel cells applications, in addition to their more common use as catalysts in the Water Gas Shift reaction. The usefulness of the O/W microemulsion reaction method for the synthesis of homogeneous mixed oxides has been demonstrated.

## Figures and Tables

**Table 1:** Temperature range of microemulsion formation and temperature used for reactions, as a function of Cu/Ce molar ratio. The surfactant/oil (S/O) weight ratio was kept constant at 25/75 and the oil phase concentration was 14 wt % (solution of isooctane and metal precursor)—ME: Microemulsion.

Molar Ratio Cu/Ce	ME Temperature Range (°C)	Reaction Temperature (°C)
0/0	24–26	-
0/100	26–28	27
5/95	26–29	27.5
10/90	26–29	27.5
20/80	27–29	28
30/70	28–30	29
35/65	28–30	29
40/60	29–31	30
50/50	29–31	30

**Table 2:** Crystallite size of Cu/Ce mixed oxide nanoparticles estimated using the Scherrer equation as a function of Cu/Ce molar ratio (as obtained samples).

Sample Cu/Ce Molar Ratio	Crystallite Size (*d_XRD_* in nm)	2θ (°)
CeO_2_	3.1	28.71
Cu/Ce 5/95	2.9	28.73
Cu/Ce 10/90	2.5	28.88
Cu/Ce 20/80	2.4	28.93
Cu/Ce 30/70	2.1	29.12
Cu/Ce 35/65	1.8	29.35
Cu/Ce 40/60	2.1	29.30
Cu/Ce 50/50	2.1	29.30

**Table 3:** Crystallite size of the calcined nanomaterials calculated by Scherrer equation as a function of Cu/Ce molar ratio.

Cu/Ce Molar Ratio Calcination Temperature	Crystallite Size (*d*_XRD_ in nm)	*2*θ (°)
Cu/Ce 20/80 500 °C	5.2	28.76
Cu/Ce 30/70 500 °C	6.9	28.74
Cu/Ce 20/80 400 °C	4.4	28.77
Cu/Ce 30/70 400 °C	4.3	28.75

**Table 4:** *E_g_* values for Cu/Ce samples obtained by Kubelka Munk function.

Material	Band Gap (eV)
CeO_2_	2.91
CuCe 5/95	2.85
CuCe 10/90	2.75
CuCe 20/80	2.67
CuCe 30/70	2.65
CuCe 35/65	2.27

## Supplementary Materials

The following are available online at www.mdpi.com/1996-1944/9/6/480/s1.
