# Supplementary material for: Synthesis of Mixed Cu/Ce Oxide Nanoparticles by the Oil-in-Water Microemulsion Reaction Method
Source: Materials (Basel). 2016 Jun 16;9(6):480. doi: 10.3390/ma9060480 (PMC5456763; doi:10.3390/ma9060480)
Supplement: Supplementary file 1 [file materials-09-00480-s001.pdf]

Supplementary Materials: Synthesis of Mixed Cu/Ce Oxide Nanoparticles by the Oil-in-Water Microemulsion Reaction Method

Kelly Pemartin-Biernath, Andrea V. Vela-González, Maira B. Moreno-Trejo, César Leyva-Porras, Iván E. Castañeda-Reyna, Isaías Juárez-Ramírez, Conxita Solans and Margarita Sánchez-Domínguez

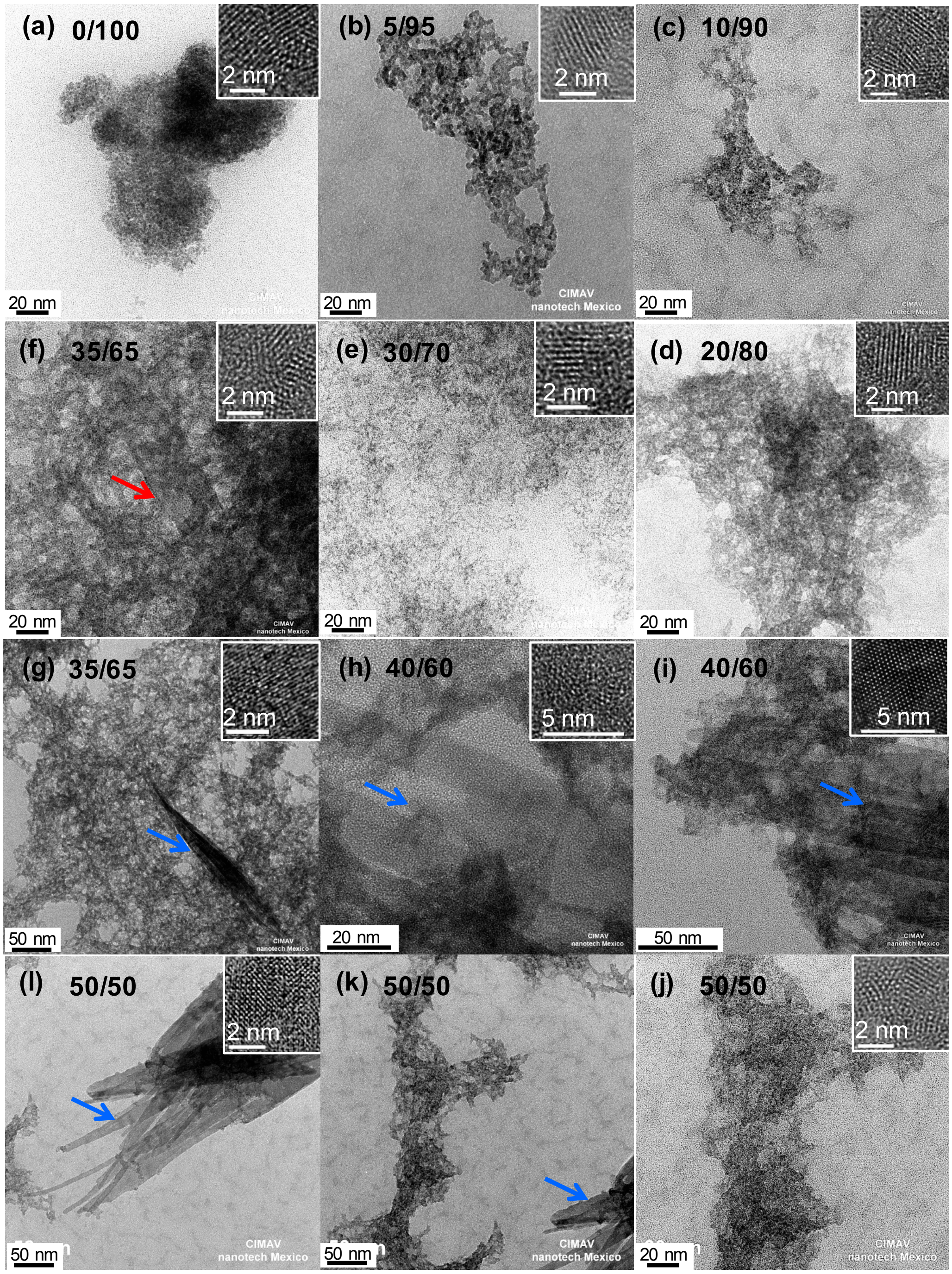

Figure S1. TEM micrographs of the synthesized nanomaterials (as obtained): (**a**) CeO_2; (**b**) $Cu_{0.05}Ce_{0.95}O_{2-\delta}$; (**c**) $Cu_{0.10}Ce_{0.90}O_{2-\delta}$; (**d**) $Cu_{0.20}Ce_{0.80}O_{2-\delta}$; (**e**) $Cu_{0.30}Ce_{0.70}O_{2-\delta}$; (**f**,**g**) $Cu_{0.35}Ce_{0.65}O_{2-\delta}$; (**h**,**i**) $Cu_{0.40}Ce_{0.60}O_{2-\delta}$; (**j**,**k**,**l**) $Cu_{0.50}Ce_{0.50}O_{2-\delta}$. A general view of the samples (low magnification) is shown, insets show a zoom of selected particles, and arrows indicate CuO nanotapes (red arrow) or assembled nanotapes (blue arrows).

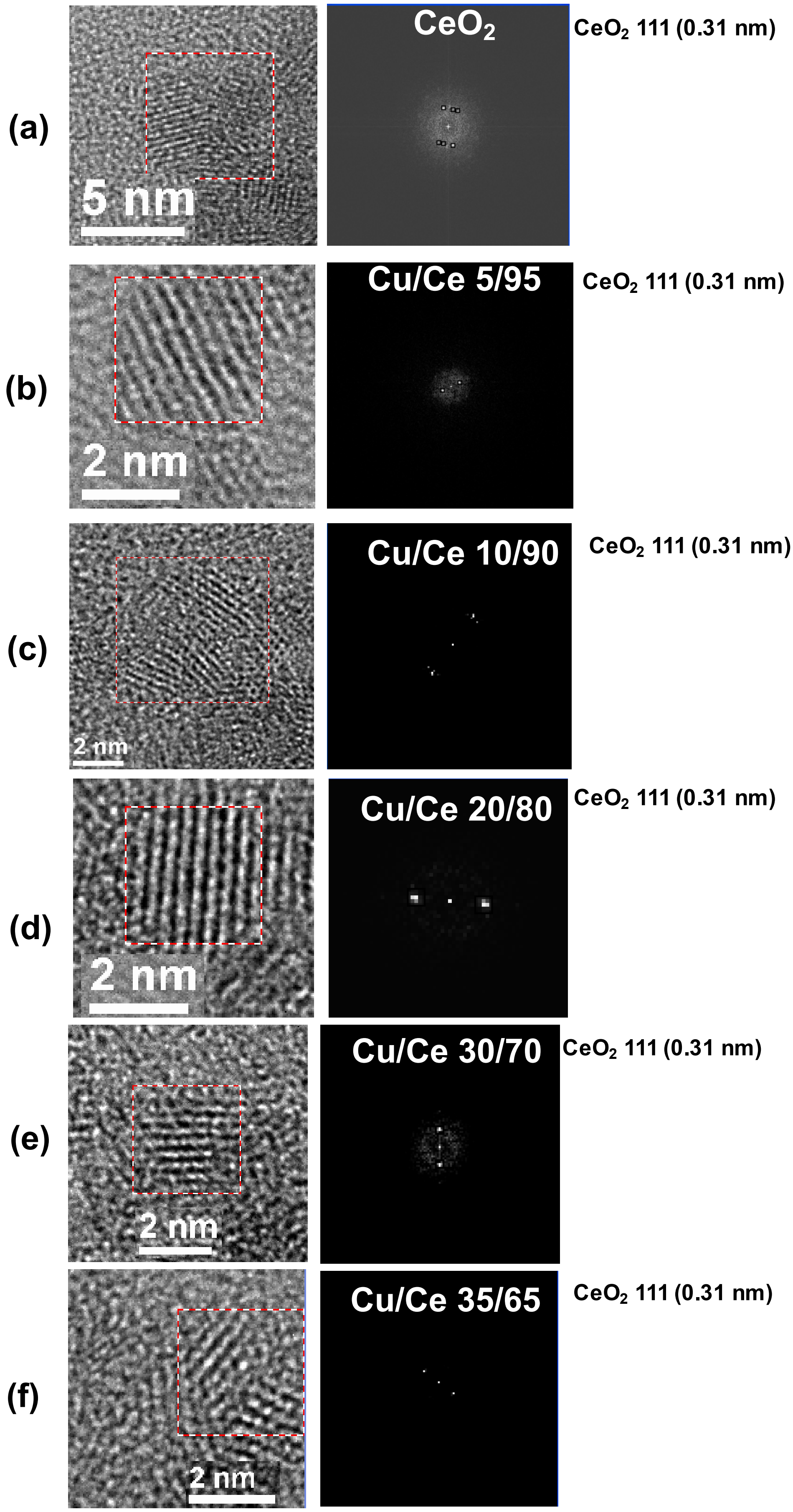

Figure S2. *Cont.*

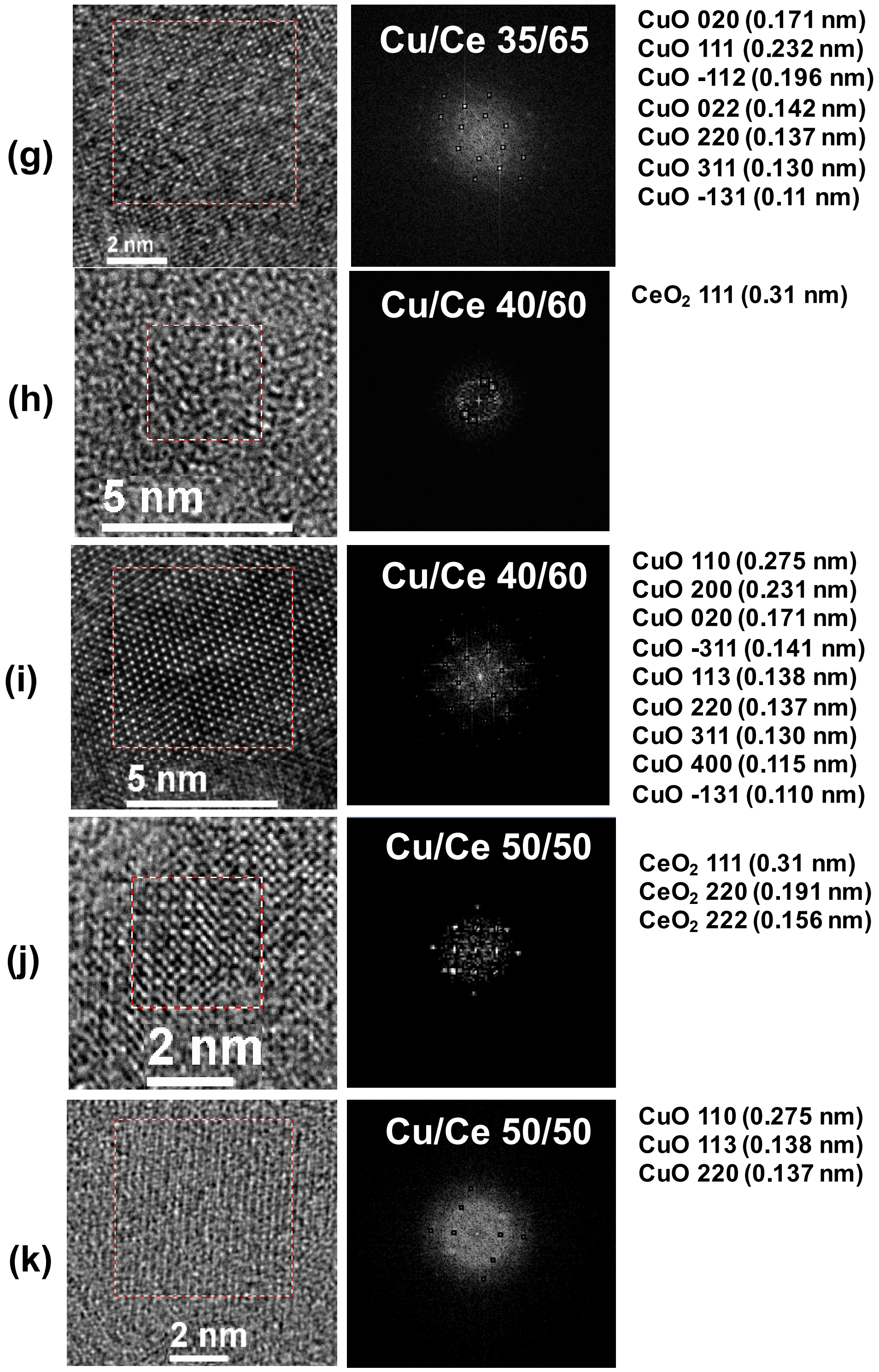

Figure S2. High resolution TEM micrographs of the synthesized nanomaterials (as obtained), with the corresponding FFT analysis of the region inside the red square, as well as the d-spacings and corresponding hkl. (**a**) CeO_2; (**b**) $Cu_{0.05}Ce_{0.95}O_{2-\delta}$; (**c**) $Cu_{0.10}Ce_{0.90}O_{2-\delta}$; (**d**) $Cu_{0.20}Ce_{0.80}O_{2-\delta}$; (**e**) $Cu_{0.30}Ce_{0.70}O_{2-\delta}$; (**f**,**g**) $Cu_{0.35}Ce_{0.65}O_{2-\delta}$; (**h**,**i**) $Cu_{0.40}Ce_{0.60}O_{2-\delta}$; (**j**,**k**) $Cu_{0.50}Ce_{0.50}O_{2-\delta}$.
